# Materials for Dentoalveolar Bioprinting: Current State of the Art

**DOI:** 10.3390/biomedicines10010071

**Published:** 2021-12-30

**Authors:** Mehdi Salar Amoli, Mostafa EzEldeen, Reinhilde Jacobs, Veerle Bloemen

**Affiliations:** 1 Surface and Interface Engineered Materials (SIEM), Campus Group T, KU Leuven, Andreas Vesaliusstraat 13, 3000 Leuven, Belgium; mehdi.salaramoli@kuleuven.be; 2 OMFS IMPATH Research Group, Department of Imaging and Pathology, Faculty of Medicine, KU Leuven and Oral and Maxillofacial Surgery, University Hospitals Leuven, Kapucijnenvoer 33, 3000 Leuven, Belgium; mostafa.ezeldeen@kuleuven.be (M.E.); reinhilde.jacobs@kuleuven.be (R.J.); 3 Department of Oral Health Sciences, KU Leuven and Paediatric Dentistry and Special Dental Care, University Hospitals Leuven, Kapucijnenvoer 33, 3000 Leuven, Belgium; 4 Department of Dental Medicine, Karolinska Institutet, SE-171 77 Stockholm, Sweden; 5 Prometheus, Division of Skeletal Tissue Engineering, KU Leuven, Herestraat 49, 3000 Leuven, Belgium

**Keywords:** bioink, hydrogel, pulp-dentin, alveolar bone, periodontal ligament

## Abstract

Although current treatments can successfully address a wide range of complications in the dentoalveolar region, they often still suffer from drawbacks and limitations, resulting in sub-optimal treatments for specific problems. In recent decades, significant progress has been made in the field of tissue engineering, aiming at restoring damaged tissues via a regenerative approach. Yet, the translation into a clinical product is still challenging. Novel technologies such as bioprinting have been developed to solve some of the shortcomings faced in traditional tissue engineering approaches. Using automated bioprinting techniques allows for precise placement of cells and biological molecules and for geometrical patient-specific design of produced biological scaffolds. Recently, bioprinting has also been introduced into the field of dentoalveolar tissue engineering. However, the choice of a suitable material to encapsulate cells in the development of so-called bioinks for bioprinting dentoalveolar tissues is still a challenge, considering the heterogeneity of these tissues and the range of properties they possess. This review, therefore, aims to provide an overview of the current state of the art by discussing the progress of the research on materials used for dentoalveolar bioprinting, highlighting the advantages and shortcomings of current approaches and considering opportunities for further research.

## 1. Introduction

Oral health is considered an integrated part of general health [1], posing a major health burden for many countries [2]. Complications arising from a lack of regenerative capacity can affect a range of dentoalveolar tissues. For example, traumas, congenital abnormalities, or tumors can lead to alveolar bone defects [3]. Moreover, periodontitis, a condition that can lead to bone resorption and eventually tooth loss [4], is considered the sixth most common human disease with a high prevalence of 45–50% [5]. Apart from periodontitis, missing teeth can also be caused by dental trauma, caries, pulp necrosis, tooth agenesis, tumor resection, and/or cyst removal [6]. While inequities still exist in the burden of oral disease worldwide, the prevalence rate of elderly people (65–74 years) having lost all their natural teeth varies from 5% to 51% in Europe, undermining the patients quality of life significantly [7].

While there are ample treatment options addressing such complications, there may be a few constraints. Available treatments for periodontitis are time-consuming, sub-optimal in the restoration of lost tissue, affected by pre-existing conditions, and most importantly, unsuccessful in 20–30% of cases [4]. Moreover, the current gold standard for treatment of complete tooth loss is the use of dental implants. Apart from dealing with a non-biological replacement, there is a specific problem caused by an age-related aspect hampering implant placement in children, where the osseointegration of the implant may prevent the natural process of concomitant bone growth with tooth eruption [8]. While techniques such as tooth autotransplantation and autotransplantation based on cone-beam computed tomography (CBCT), involving surgical repositioning of a tooth from one position to another, have addressed the issue to some extent, challenges such as correct selection of the patient with regards to age, donor tooth and root length, presence of suitable donor and recipient sites, presence of sufficient alveolar bone support, and the requirement of tooth extraction limit its use [9,10,11,12,13].

The challenges associated with current treatment options have led to the exploration of regenerative strategies, aiming to develop biological substitutes that can restore, maintain, or improve the function of the tissues [14]. In the field of tissue engineering, a subfield of regenerative medicine, the aim is to fabricate such a tissue analogue by combining principles of life sciences and engineering [15]. This could potentially offer a sustainable solution to complications such as alveolar ridge resorption, tooth loss, condylar resorption, and craniofacial defects.

Several tissue engineering approaches have been explored for the regeneration of dentoalveolar tissues. As a first step, regeneration of pulp-dentin complex has been the focus [16] by using scaffolds based on natural polymers such as collagen [17], synthetic polymers such as poly lactic acid (PLA) [18], or through injectable hydrogels [19]. Other strategies have been implemented to regenerate periodontium tissues, including the incorporation of guided tissue/bone regeneration membranes [20], and several natural/synthetic scaffolds or transplantation strategies have been explored for the regeneration of impacted bone [21]. While these efforts have been promising, there are still challenges associated with low cell engraftment, inaccurate localization of the cells, the possibility of immunological rejection, the difficulty of delivering required growth factors efficiently, the inability to control the type of formed tissue, a possible shortage of cells in the defect area and lack of microvasculature formation [20,21,22,23,24,25].

However, the main challenge arises from the fact that the dentoalveolar complex is a hybrid organ composition made of highly specialized nerves and mechanoreceptors, ligaments, enamel, dentin, root, cementum, and bone. Current regenerative practices are focused on regenerating individual tissues, and thus, they fail to mimic and regenerate such complex tissue architectures.

**Figure 1:**
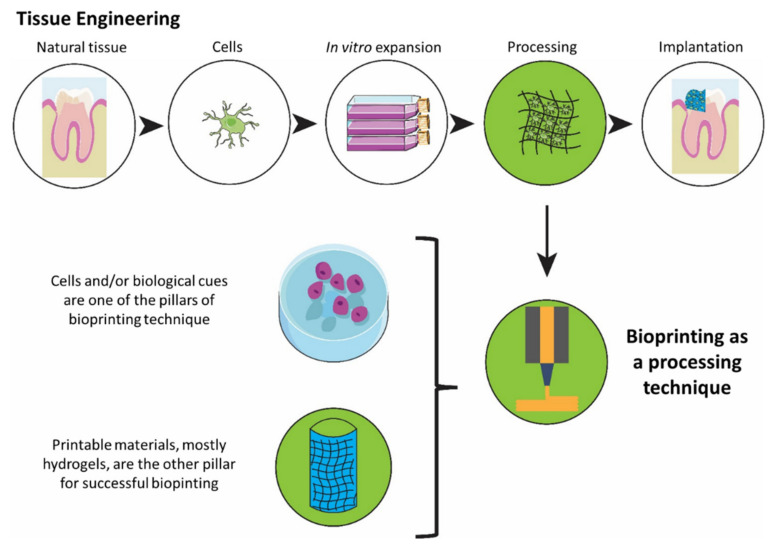
An overview of a tissue engineering process highlighting bioprinting as a promising fabrication technique.

Aiming to potentially solve some of these challenges, bioprinting is an emerging technique that allows the production of patient-specific implants through an automated process of fabrication, enhancing the ability to control cell positioning and increasing the possibility of upscaling while improving the outcome by reducing the variability, and increasing robustness. Bioinks combine cells and/or biological cues such as growth factors with printable scaffolding material, mainly based on hydrogels. The hydrogels used for printing are deposited through a nozzle mainly in an extrusion, inkjet, or laser-based process. Upon deposition on the substrate, they are solidified through polymerization, producing a cell encapsulated construct [26]. A schematic overview of tissue engineering and the role of bioprinting in the process is presented in Figure 1.

Starting from the design retrieved from computed tomography (CT), cone-beam computed tomography (CBCT), or magnetic resonance imaging (MRI), bioprinting presents the opportunity to produce patient-specific constructs addressing specific needs of individuals, and facilities direct placement of different biomolecules and scaffold material in desired locations [27]. Additionally, the possibility to use different bioinks in one process enhances the ability to mimic the complexities of the natural tissue. This ability has been represented in bioprinting of vasculature using coaxial nozzles, and carbon nanotube reinforced alginate [28] or by using sacrificial Pluronic F127 [29], in bioprinting of nerve conduits using novel stereolithographic techniques [30], or in the development of liver models for drug testing [31].

**Figure 2:**
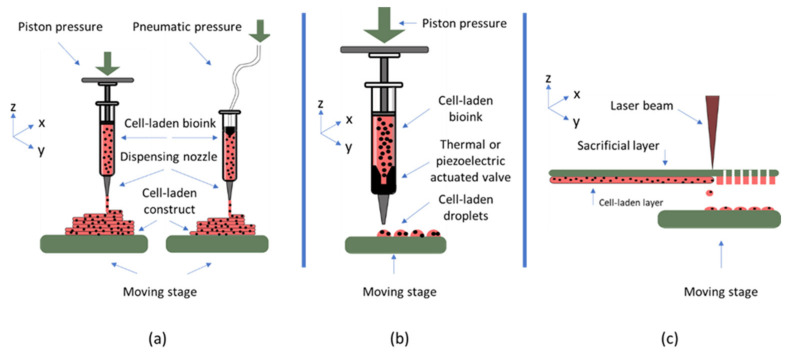
Most widespread bioprinting technologies are (**a**) extrusion-based bioprinting (**b**) inkjet bioprinting, and (**c**) laser-based bioprinting.

While a wide variety of different bioprinting techniques have been developed, most widespread bioprinting technologies include extrusion, inkjet, or laser-based bioprinting. In the extrusion process, a filament of material is formed, which will be placed on the substrate forming the 3D construct. In inkjet and laser-based printing, droplets of cell encapsulating material are produced and deposited on the substrate through different methods. This represents different requirements in the materials used for these techniques, as presented in Table 1 and in the requirements section. A schematic representation of the main bioprinting technologies is presented in Figure 2.

As bioprinting relies on cell and growth factor encapsulated biomaterials, the choice of these materials has a significant impact on the outcome of the tissue engineering process. However, while there are some studies discussing the materials used for 3D printing of scaffolds for tissue engineering in this region, so far, there is no study focused on reviewing the materials used for the preparation of cell-encapsulating bioinks for bioprinting strategies in the dentoalveolar region. Considering the importance of bioprinting as a new technology in the field of dentoalveolar tissue regeneration, and the influence of choosing an appropriate material for specific tissues, along with the lack of a comprehensive review focused on this topic, this review aims at providing an overview of the work that has been done in this context, along with the requirements and research that could potentially evolve to develop reliable bioprinting strategies for mentioned tissues. Consequently, first, the requirements for choosing the appropriate material will be discussed, followed by different classes of materials that have been used or can be used for bioink development for the dentoalveolar tissues, highlighting the possibilities and limitations of each, along with presenting possible areas of research in this field that remain unexplored.

## 2. Bioink Requirements for Dentoalveolar Tissue Engineering

### 2.1. General Requirements for a Bioink Material

Bioinks are usually biomaterials encapsulating cells, which will then be deposited on a substrate to form three-dimensional constructs. Consequently, biomaterials used for this purpose must possess a range of biological and physicochemical properties, depending on the target tissue and the bioprinting modality used. In general, these materials are in direct contact with the cells. Therefore, one of the most important qualities they must possess is biocompatibility. In the context of bioprinting, biocompatibility encompasses both absences of cell toxicity and the presence of cell adhesion cues. This is significantly influenced by the water content, facilitating the transfer of oxygen and nutrients to the cells [53]. Furthermore, these materials need to be biodegradable, as they need to be replaced by extracellular matrix components produced by the cells. As a result, it is important for the degradation profile to match the regeneration rat extracellular matrix (ECM) by the cells [54]. Furthermore, mechanical properties, such as stiffness, must match those of the target tissue to provide a favorable environment for cellular activities [55]. Moreover, the possibility of rapid gelation is of extreme importance in order for the bioink to keep its shape upon deposition on the substrate [56].

In addition to these general requirements, each bioprinting modality imposes additional considerations on the choice of material. In extrusion-based bioprinting, the material needs to form a filament upon ejection from the nozzle. During the process, shear stress is applied to the cell encapsulating material introducing the possibility of damage to the cells, thus, properties such as shear thinning and yield stress of the material must be considered [57]. While in extrusion-based bioprinting the viscosity of the material should be in the range of 30 to 60 × 10^7^ mPa/s, the viscosity in inkjet technique has to be much lower and in the range of 3.5 to 12 mPa/s [58]. This is because, in this technique, different methods such as thermal or piezoelectric actuation are used to produce droplets of the material, rather than filaments. Furthermore, bioinks for this printing modality need to have rheopectic properties rather than shear-thinning properties, meaning there needs to be an increase in viscosity upon application of shear force, leading to droplet formation. As for laser-based bioprinting, the bioink should be able to adhere sufficiently to the sacrificial layer and spread uniformly on it. Additionally, it needs to possess high viscoelasticity, enabling formation of material jets. The viscosity of the material used for this bioprinting modality is usually in the range of 1 to 300 mPa/s [58].

As the target tissue of the regeneration process has a significant effect on the requirements of the bioink, and the modality is chosen, the main characteristics of most important dentoalveolar tissues and required material properties for each tissue are described in following sections.

### 2.2. Requirements of Bioinks for Dental Pulp Regeneration

Dental pulp is a connective tissue composed of cells embedded in a collagen-based ECM, 75% of which is consisted of water, hence, encouraging the use of hydrogels as tissue engineering scaffolds. The pulp core, containing fibroblasts, is surrounded by a cell-rich zone accommodating dental pulp stem cells (DSPCs) along with fibroblasts. Encompassing this cell-rich zone, there is a cell-free zone housing the capillary network along with nerves. Finally, in the interface with dentin lies the peripheral area of the pulp containing odontoblasts, differentiated from the stem cells in the cell-rich zone [59,60]. This heterogeneity within the cell types present in dental pulp must be taken into account when designing a bioink for this tissue. The most important parameter to consider in this regard would be the localized differentiation of the DPSCs in the interface with dentin, and the presence of undifferentiated stem cells in the core [48].

Furthermore, the pulp tissue, consisting of collagen fibers, proteoglycans and cells, is the only soft tissue inside the tooth. This tissue exhibits a gel-like linear viscoelastic behavior with a storage modulus of approximately 100 Pa and a loss modulus of around 10 Pa. This tissue represents a young’s modulus of 0.8 ± 0.4 kPa and a toughness value of 37.7 ± 19.1 kPa, along with a time-dependent stress relaxation caused by the viscoelasticity [61]. These mechanical properties have been known to have a significant effect on activities of DSPCs such as proliferation and differentiation, enabling control over cellular behavior in the tissue engineering construct [62]. Consequently, it is important for the hydrogels used for bioprinting strategies to represent these mechanical properties to create a favorable environment for the native cells to function and regenerate natural tissue.

### 2.3. Requirements of Bioinks for Dentin

Odontoblasts present in the outer interface of the pulp have the principal function of producing dentin during tissue formation and tissue regeneration. Consequently, provided a favorable environment, stem cells in the dental pulp are capable of regenerating dentin tissue. However, this regeneration process could be enhanced by the use of scaffolds, and bioprinting enables the production of scaffolds closely resembling the complex structure of dentin. Dentin itself is a mineralized, tubular structure surrounding the pulp, and it is composed mainly of hydroxyapatite crystals [63]. It has a very complex structure with a range of properties depending on the location, the alignment of microtubules and the density of mineralization, with a Young’s modulus ranging from 17 GPa in regions close to the pulp, to 42 GPa in center regions [64].

While the dentin is deposited by odontoblasts, the microenvironment is known to have a significant effect on behavior and activity of these cells. Consequently, replication of the tubules, their structure, and their mechanical properties can have a significant effect on the success of the tissue engineering process [65]. For example, the microporosity of the material must allow the cells to extend their cytoplasm on the surface and regenerate the tissue. A pore size of approximately 300 μm is known to provide sufficient space for odontoblasts to regenerate dentin-like tissue [66].

### 2.4. Requirements of Bioinks for Periodontal Ligament Bioprinting

Periodontal ligament (PDL) is a specialized and vascularized soft connective tissue, mainly composed of collagen fibers, surrounding the tooth and filling the space between the tooth and its socket in the alveolar bone. PDL is tasked with supporting the dental structure, providing sensory information and assisting in the regeneration of damaged tissue, as well as with maintaining homeostasis in osteogenesis of alveolar bone. A wide variety of cell types are present in PDL, including fibroblasts, bone-related cells such as osteoblasts and osteoclasts, cementoblasts and cementoclasts, and periodontal ligament stem cells (PDLSCs), which are used widely for tissue engineering of PDL and other dentoalveolar tissues [67].

The inherent complexity of the PDL tissue and its behavior in reaction to stress, along with differences in characterization techniques, has resulted in a range of analytical and experimental models to analyze the mechanical behavior of the material. These include assumption of PDL as an isotropic homogeneous tissue with linear elastic properties, hyperelastic models, and viscoelastic models describing behaviors such as force-relaxation and hysteresis [68,69]. However, the non-linear time dependency of the PDL behavior with regard to tooth movement has been shown in several experimental studies [70]. While there are discrepancies in the reported literature with regard to the physical and mechanical properties of PDL [68,69], from a tissue engineering standpoint, the most important requirement is to ensure the regeneration of functional support for the tooth, and to provide the proper environment for the stem cells to enable this regeneration.

For this purpose, time-dependent viscoelasticity has been reported to best reflect the mechanical behavior of natural PDL. Such models have been developed and discussed in detail in various studies [71,72,73] and a Young’s modulus of 5 × 10^6^ N/m^2^ and a Poisson’s ratio of 0.45 have been experimentally detected for the PDL [74]. These properties such as mechanical loading and stiffness are known to have a significant effect in the modulation of PDLSCs behaviors such as proliferation and differentiation [75], with both proliferation and osteogenic differentiation of the cells increasing with increased stiffness in a range of 6 to 135 kPa [76]. Consequently, being mindful of these properties in a bioink is extremely important to control the behavior of encapsulated or invading cells and the regeneration of a functional tissue.

### 2.5. Requirements of Bioinks for Alveolar Bone Bioprinting

Alveolar bone is a flexible ridge of the jaw that incorporates tooth sockets and supports the dental structures. Alveolar bone is made up of four main layers, and their differences should be taken into account in any tissue engineering effort aimed at these tissues. These layers are the periosteum, compact radiodense bone, cancellous bone, and the cribriform plate housing tooth sockets [77]. This structure easily responds to different stimuli such as tooth loss, usually in the form of resorption, which can cause major challenges in other treatment strategies such as implant placement. In fact, the structure of the alveolar bone along with the cell behavior in the region has led to a higher responsiveness compared to other bones in the skeletal system such as long bones [78]. Consequently, bioprinting can be significantly helpful in the recapitulation of such a complex structure leading to the regeneration of tissue analogues. Similar to other bones in the body, alveolar bone is highly mineralized with the majority of mineral content being semi-crystalline hydroxyapatite. Furthermore, 70% of its structure is composed of an organic matrix, the main component of which is collagen type I. It includes 2 to 5% cells, mostly being bone-lining cells, osteoblasts, osteoclasts and osteocytes [79].

Tissue engineering of the bones in the alveolar region requires consideration of their differences with other bones in the body in terms of their functional activities, including the load applied during physical activities of the face [80]. Consequently, the bioink needs to support the bioactivity of different cell types present and account for differences in the mechanical properties of different layers of the bone. The most important mechanical property to replicate is the Young’s modulus, which is approximately 13.7 × 10^9^ N/m^2^ in high-density alveolar bone and close to 0.9 × 10^9^ N/m^2^ in cancellous alveolar bone [74,81]. Moreover, a pore size of 150–500 μm has been known to facilitate bone tissue regeneration and formation of the vasculature [82]. While bioprinting can facilitate fabrication of cell encapsulated scaffolds for the complex architecture of alveolar bone, the possibility of directing stem cells towards tissue regeneration, especially considering that the most widely used materials for bioprinting are hydrogels of weak mechanical properties, remains a challenge which needs to be addressed by the specific design of bioinks, along with the dynamic processes involved in the tissue regeneration [83].

## 3. Bioinks

Hydrogels, a class of materials comprised of vast, water-encapsulating polymeric networks, are the most common class of materials used for bioprinting. This is due to their favorable properties, which could meet the general requirements for a bioink, including large water content resembling natural ECM, support of cell attachment, growth and differentiation, in addition to biodegradability and viscoelastic properties [84,85]. Hydrogels used for bioprinting in the dentoalveolar region can be divided into two main categories, hydrogels based on natural polymers and synthetic polymers. Natural hydrogels used in this field could be further classified into hydrogels based on collagen, hyaluronic acid, fibrinogen and alginate. An overview of the materials used for bioprinting dentoalveolar tissues is presented in Table 1, and the classes of materials are discussed in detail in this section.

### 3.1. Natural Polymers

#### 3.1.1. Collagen-Based Materials

Collagen is one of the main components of the natural ECM, consequently, it has an excellent biocompatibility making it a very interesting option for different tissue engineering strategies. Moreover, it can form hydrogels at 37 °C, making it possible to encapsulate cells. In the dentoalveolar field, dental pulp stem cells (DSPCs) encapsulated in collagen type I and II have shown viability of over 95% [86]. However, low mechanical properties have made it essential to explore the possibility of combination with other materials, crosslinking or performing modifications on natural collagen in order to use it for bioprinting [87]. Through such modifications, collagen has been widely used in inkjet [88], extrusion-based [87], and laser-based [89] bioprinting for the regeneration of different tissues including skin [90], bone, cartilage [91], cardiovascular [92], liver [93] and nervous tissues [94], in addition to the dentoalveolar tissues.

In dentoalveolar bioprinting, agarose was used by Campus et al., to increase the viscosity, and enhance the mechanical properties of collagen for inkjet bioprinting of alveolar bone, enabling bioprinting of complex structures. While the increased agarose content reduced cell spreading, the increased stiffness resulted in a higher differentiation of mesenchymal stem cells [40]. However, a compressive modulus ranging from 18.1 ± 3.5 kPa to 89.1 ± 13.9 kPa, compared to natural bones 110 MPa, could potentially lead to complications in the integration of the construct in the implantation stage, especially in load-bearing regions [40,95], highlighting the need to consider incorporation of mechanically stronger materials such as thermoplastic polymers [96]. However, in a subsequent study, the same material, plus fibrinogen, was used for bioprinting of dental pulp [51]. The mechanical properties obtained for the similar material [40], suggest the properties to be more suitable for regeneration of dental pulp than bone. Still, a storage and loss modulus of approximately 280 and 20 Pa, respectively, are significantly higher than those of natural tissue, demonstrating room for improvement of the bioink in terms of physical properties. Interestingly, a co-culture of DSPCs and human umbilical vein endothelial cells (HUVECs) resulted in the formation of microvessels in the bioprinted structures within 14 days of in vitro culture, a very important requirement for regeneration approaches directed towards dental pulp [51].

While collagen in its native form is being used widely for bioprinting and tissue engineering strategies, the denatured form of the protein derived from its hydrolysis, gelatin, is used much more extensively. Retaining the RGD sequences present on collagen, gelatin is also highly biocompatible, in addition to the fact that it does not trigger an immune response and it is more cost-effective than collagen [97]. Furthermore, the need for a rapid and efficient crosslinking in the field of bioprinting has given rise to the use of a gelatin derivative called gelMA, or gelatin functionalized with methacryloyl groups [98]. The possibility of encapsulating mouse dental pulp cells (OD-21) in gelMA has led to viabilities of over 80% [99] and 90% [100] in different studies. The reduced viability as compared to collagen can be attributed to the presence of photoinitiators, toxic by nature, and UV irradiation, as it was determined that increasing the crosslinking time could reduce the viability [99]. Furthermore, similar to collagen, encapsulation of DSPCs and HUVECs in the material has resulted in the formation of vasculature [101].

Beyond only cell encapsulation, a combination of gelMA and poly(ethylene glycol) dimethacrylate (PEGDA) has been used in inkjet bioprinting for regeneration of periodontal tissue. While the incorporation of PEG resulted in enhanced control over droplet size, PDLSCs encapsulated in this material demonstrated higher viability and spreading in higher gelMA concentrations [45], the same tradeoff observed in collagen combinations, which could potentially be addressed by incorporation of cell-binding motifs to account for lack of bioactivity on PEG [102]. Further properties of this bioink were analyzed in a study aimed at regeneration of alveolar bone, where stiffness in the range of 4.5 kPa to 23.5 kPa was observed, and in vivo analysis resulted in bone formation within 6 weeks of implantation [37]. Still, a lack of further in-depth characterizations and the relatively rapid degradation of around 80% in two weeks leads to questions about structural integrity in the long term, showing room for further research.

In addition to inkjet, gelMA has also been used as the base material for extrusion-based bioprinting strategies in the dentoalveolar region. For this application, optimization of parameters such as polymer concentration, photoinitiator type and concentration, UV exposure time and printing parameters can have a significant effect on the outcome, as shown by Raveendran et al. [44]. However, limitations imposed by the parameters, such as polymer concentration, lead to the need for exploration of complementary strategies, such as the utilization of polymeric support rather than increasing polymer concentration, as shown in the study by Kuss et al., where the incorporation of polycaprolactone/hydroxyapatite resulted in a compressive modulus of around 50 MPa, much closer to natural bone than pure hydrogel [38]. In addition to mechanical properties, strategies to induce angiogenesis are essential for the regeneration of functional tissues. While some studies have suggested a co-culture of HUVECs and region-specific stem cells [51,101], post-processing by subjecting bioprinted constructs to short-term hypoxia was shown to be effective by Kuss et al. [38]. A comparison of these methods could potentially lead to a better understanding of the angiogenesis within the engineered constructs.

#### 3.1.2. Materials Based on Hyaluronic Acid

Hyaluronic acid (HA) is a glycosaminoglycan (GAG) abundant in the ECM of many soft tissues. As a result, it is biocompatible, biodegradable, does not trigger an immune response and is non-thrombogenic. Possessing shear thinning properties in addition to these, hyaluronic acid has been used extensively in the field of tissue engineering and bioprinting [103]. However, similar to other natural-based hydrogels, hyaluronic acid suffers from weak mechanical properties making it difficult to print stable constructs. Consequently, crosslinking or chemical modification is often required to ensure long-lasting mechanical integrity, post-printing shape fidelity, and controlled degradation behavior [53]. Such modifications have enabled the use of hyaluronic acid in bioprinting of bone, cartilage, vascular tissue, amongst others [104,105].

In the field of dentoalveolar regeneration, materials based on hyaluronic acid have been used as cell-encapsulating hydrogels for regeneration of both alveolar bone and pulp tissue with different strategies to augment its mechanical properties. Combining hyaluronic acid with a thiol derivative of gelatin (gelatin-DTPH) and crosslinking with polyethylene glycol diacrylate, along with the use of polycaprolactone as support, has been proposed for alveolar bone regeneration. The encapsulation did prove suitable as mineral deposition was observed within four weeks of in vivo implantation [106]. However, the lack of any physical and mechanical characterizations makes it difficult to assess the role of different constituents of the material in cellular behavior. In a different strategy, a combination of hydrazide-modified hyaluronic acid with cellulose nanocrystals resulted in crosslinking between the functional groups of the two materials, enhancing material stability. Encapsulation of both DSPCs and HUVECs leads to formation of blood vessels within two weeks of in vitro culture [107]. This, along with a reported gelation time of 3 to 30 s makes the material a suitable option to be explored in bioprinting strategies as well. However, with a full degradation being achieved within 2 to 13 days, further research and optimization of the material might be required to increase its stability.

The step towards bioprinting using hyaluronic acid was first taken by combining hyaluronic acid with gelatin, fibrinogen and glycerol to produce a bioink for extrusion-based bioprinting aimed at alveolar bone regeneration, where the cell-laden hydrogel would be printed along with polycaprolactone support. Furthermore, use of sacrificial material, Pluronic F127, enhancing the precision of the printing was first reported [39]. The development of such a system encompassing the cell encapsulated hydrogel, mechanical support and sacrificial layers could be considered a major step towards the development of clinically relevant tissue engineering constructs through bioprinting. In a strategy different from combining HA with other materials, modification of hyaluronic acid by methacryloyl groups and demonstrating its potential to be used along with gelMA and polycaprolactone for alveolar bone bioprinting was a major innovation in the study by Kuss et al. [38], showing the importance of slight chemical modifications and use of hyaluronic acid derivatives in the success of bioprinting strategies.

#### 3.1.3. Fibrin-Based Materials

Fibrin is a glycoprotein consisting of fibrinogen monomers, and is known to have a critical role in blood clotting, inflammatory responses and wound healing. Consequently, fibrin can be a suitable candidate to act as an active scaffold enhancing cell adhesion and proliferation [108], and its hydrogels have been widely used in tissue engineering of different tissues [109] as well as in bioprinting strategies aimed at regeneration of nervous tissue [110], skin [111], bone [112], vascular tissue [113], and dental tissue [48]. Fibrin has been reported to support differentiation of DSPCs into odontoblasts, and vascularization of dental tissues, making it a promising candidate for dentoalveolar tissue engineering [48]. However, challenges such as gel shrinkage, low mechanical stability and rapid degradation are faced in tissue engineering strategies using fibrin. To circumvent this, modification of fibrin and synthesis of polyethylene glycol fibrinogen was one strategy resulting in the viability of over 85% among encapsulated DPSCs. However, the storage modulus ranging from 140 Pa in pure fibrin gel to 3601 Pa in maximum PEG concentration is significantly higher than that of natural dental pulp [114], risking the possibility of full mineralization and bone formation rather than the regeneration of a functioning pulp tissue.

As mentioned in the requirements section, one of the most critical aspects of pulp-dentin tissue regeneration is the localized differentiation of DSPCs to odontoblasts at the interface of the pulp-dentin. A gradient of fibrin concentrations was used by Han et al. [48], leading to a compressive modulus ranging from 270 Pa to 400 Pa. As a result, differentiation to odontoblasts only happened in higher concentrations of fibrin, enabling the possibility of accomplishing localized differentiation of the DPSCs. While this achievement is a major step forward in the development of bioinks for pulp-dentin regeneration, challenges such as the internal pores size of 2 to 4 µm, which could negatively affect angiogenesis and tissue formation [115], or the degradation rate of 30 to 60% within 5 days, demonstrate the need for further optimization of the suggested bioink.

#### 3.1.4. Alginate-Based Materials

Alginate is a biocompatible, non-immunogenic natural polymer capable of dissolving in water and forming hydrogels through ionic crosslinking, which has become very popular in the field of bioprinting. This interest is mainly due to the fact that the material properties such as compressive modulus, stiffness, or gelation rate can be easily modified by optimizing the polymer density and crosslinking density [116]. However, the main disadvantage of alginate is its lack of biological cues preventing cells from adhering to the alginate, leading to cell death. Several methods have been suggested to address this issue, including the combination with other materials, or modification with cell-binding peptides such as the incorporation of RGD peptides or laminin sequences on its backbone [117]. For example, Bhoj et al. [118] made alginate cell-friendly through modification with RGD sequences. Encapsulation of human umbilical cord stem cells and DSPCs in the gel resulted in high cell viability and proliferation, presenting an injectable scaffold capable of regenerating pulp-like tissue [118]. A different strategy was suggested by Sevari et al. [119], who combined alginate with Matrigel and bioglass for the regeneration of craniofacial tissues. As expected, encapsulation of DSPCs demonstrated that a higher Matrigel concentration leads to better cell viability [119]. The similarity of the viability data in the two studies suggests both strategies to be acceptable from a biological point of view.

The satisfactory and tunable mechanical properties have led to a wide use of alginate in the field of bioprinting tissues such as bone or cartilage [120]. Furthermore, alginate has been widely used to bioprint vasculature in regenerative strategies [121], such as the study by Yu et al., where bioprinting of hollow tubular channels using alginate printed through coaxial nozzles was demonstrated [122]. However, so far, alginate has only been used in one study for extrusion-based bioprinting of the dentin-pulp complex. To augment the cellular response, soluble dentin matrix was combined with alginate, leading to differentiation of DSPCs to odontoblasts even without the use of additional growth factors [49]. While showing the promise of using natural tissue in bioink formulations, the complicated procedure of obtaining the dentin matrix, along with limited resources of the material, may hamper the use of this bioink in upscaling strategies as a step towards clinical applications.

### 3.2. Synthetic Polymers

While natural polymers are perfect materials for tissue engineering in terms of biological response [123], the variability of physical, mechanical and biological properties from batch to batch turns the production of a reliable bioink based solely on natural polymers a difficult task. Moreover, most natural polymers suffer from low mechanical properties, reducing their reliability in the printing procedure and their capacity to regenerate hard tissues such as bone [84]. On the other hand, synthetic polymers offer a high control over different physicomechanical properties such as compressive modulus or degradation rate due to the tunable nature of their synthesis procedure, being built up from single blocks of monomers. Therefore, several synthetic polymers have been used in tissue engineering strategies to produce hydrogels with a reduced variability in their properties. However, one major challenge of most synthetic polymers is to compensate for their lack of cell adhesion sites, resulting in the need to either incorporate such molecules on the polymer backbone, or to combine them with natural polymers [124]. In the framework of bioprinting, synthetic polymers are incorporated with three main goals: (i) to enhance the physicomechanical properties of the constructs as part of the bioink, (ii) to improve the shape fidelity of printed constructs by acting as a frame for softer bioinks, and (iii) to provide extra functionalities such as for sacrificial layers [125]. Several synthetic materials have been used for such functions including poly (hydroxyethyl methacrylate) (pHEMA), poly (ethylene glycol) (PEG), polycaprolactone (PCL), Poloxamer 407 (pluronic f127), and poly (N-isopropylacrylamide) (pNIPAM).

A variety of synthetic polymers have been used to encapsulate cells for tissue engineering of dentoalveolar tissues. PEG is one of these polymers, however, it is incapable of forming 3D networks and thus, hydrogels. As a result, it requires modifications such as acrylation. PEG-diacrylate combined with fibrinogen has been used to encapsulate DSPCs with cell viability of over 85%, and a storage modulus ranging from 453 Pa to 3601 Pa. Similar to the study by Han et al. (described earlier) [48], it was determined that increased mechanical properties, could lead to odontoblastic differentiation [114]. While this study did not include printing, it could evolve into bioprinting of pulp tissue with localized differentiation based on material properties. Similarly, in a study using pNIPAM-chitosan-graphene oxide by Amiryaghoubi et al. [126], osteogenic differentiation of DSPCs was observed and attributed to the presence of graphene oxide [126], suggesting the possibility of using hydrogel composition as a trigger for differentiation behavior in the dentoalveolar regeneration approaches. Moreover, synthetic materials have been used in a number of the studies mentioned earlier in the field of bioprinting dentoalveolar tissues for different functionalities described. Polyethylene glycol diacrylate was used by Ma et al. as part of the bioink in two studies [37,45] to enhance the mechanical properties of the bioink. Polycaprolactone was used as mechanical support to stand as a scaffold for cell-encapsulated bioinks by Han et al. [48] and Kang et al. [39]. Additionally, Pluronic F127 has been used by Kang et al. as a sacrificial layer for the bioprinted constructs [39].

Overall, it is clear that while different classes of hydrogels can be used for dentoalveolar tissue regeneration, often modifications or combinations of them are required for a successful bioprinting strategy. In Table 2 and Table 3, a summary of hydrogels that have been used to encapsulate cells for dentoalveolar regeneration has been given, all of which have the potential to be used in bioprinting strategies. Furthermore, a list of the materials that have been used in regeneration of other tissues through bioprinting, and which could be applied to the dentoalveolar region using appropriate cell types and biological factors, has been provided. Further structural analysis of the mentioned polymers, and possible modifications on those have been described in the [127,128,129,130].

## 4. Discussion and Future Perspectives

The choice of materials is one of the key aspects in developing bioprinting strategies for dentoalveolar tissue regeneration. Since the alveolar region is a heterogeneous system consisting of multiple tissues with each having its own physical, mechanical and biological properties, appropriate bioinks should be considered to regenerate these tissues. A wide range of materials have been suggested to be used in bioprinting dentoalveolar tissues. While using hydrogels from natural polymers seems to be a suitable choice for bioprinting due to their support for cellular activities, requirement of mechanical strength, especially in tissues such as dentin or alveolar bone, highlight the need for further modifications and/or combinations of these polymers with synthetic polymers to achieve viable strategies for regeneration of these tissues.

Among these tissues, dental pulp is a soft, vascularized, viscoelastic tissue. Collagen and fibrin seem to be the most promising candidates among discussed materials in terms of cellular response, while in pure form, two of the materials with lowest mechanical properties. They possess viscoelastic properties that could be adjusted to match those of natural pulp tissue through adjustment of material composition and concentration [61]. For this purpose, a wide range of modifications remain unexplored in the field of dentoalveolar bioprinting. Use of collagen derivatives such as methacrylated collagen which is photocrosslinkable [167], compositing collagen with molecules such as calcium phosphates [168], crosslinking of collagen [169] or printing along with stronger thermoplastic polymers [170] are some of these modifications, in addition to exploring options to exert control over physicomechanical properties of gelMA. These options could range from blending with viscosity-enhancing materials such as gellan gum [171], relying on inherent temperature behavior of gelMA [172], or compositing gelMA with materials such as calcium phosphates [173], or polysaccharides [174], to chemical modifications of gelatin with acrylamides [175], styrene groups [176], norbornene [177], etc. Furthermore, while fibrin has been used in combination with other materials, other methods such as chemical modification or incorporation of plasmin inhibitors such as laminin, have not been studied in the dentoalveolar field yet [178,179]. Through these modifications, further control over mechanical properties of the materials could be achieved, which in turn, could lead to enhanced control over cellular processes such as differentiation, enabling achievement of localized differentiation within the scaffold, and combined with cells such as dental pulp stem cells, human umbilical cord stem cells, stem cells from apical papilla or endothelial cells, they can be of interest in research aiming to regenerate vascularized soft tissue such as dental pulp.

In addition to these, alginate and hyaluronic acid have been used extensively in the field of bioprinting, and to a lesser extent, in the bioprinting of dentoalveolar tissues. Degradation rate, viscoelastic profile and mechanical stability of hyaluronic acid can potentially be tuned by controlling the molecular weight of hyaluronic acid, Incorporating other natural or synthetic polymers such as methyl cellulose [180], dextran, polyethylene glycol, and polycaprolactone [181], modifying the material through esterification or methacrylation [182], or crosslinking the available functional groups using different modifiers such as carbodiimides [183], or Schiff-base reactions [184]. Moreover, the lack of bioprinting research using alginate in the dentoalveolar region, combined with opportunities presented by the tunability of alginate hydrogels and the feasibility of making them cell friendly, demonstrate a clear path towards further research using this material. Still, due to the soft nature of alginate and hyaluronic acid, their usage without support from synthetic polymers is limited to soft tissues in the region, where the very high tunability of the properties could benefit the complex mechanical requirements of tissues such as periodontal ligament.

Bioprinting of hard tissues in the region, such as alveolar bone, seems to require comprehensive strategies incorporating hydrogels, thermoplastic support, and sacrificial layers. The hydrogels used in the bioprinting of these hard tissues need to possess enhanced mechanical properties as compared to those used in the regeneration of soft tissues. For this purpose, strategies suggested in the previous paragraph, such as crosslinking, could be a first step to increase the stability of the hydrogels. However, further modifications and combinations, such as the incorporation of hydroxyapatite [41], calcium phosphate and calcium silicate [46], or carbon nanotubes [185] could enhance the mechanical properties and lead to osteogenic differentiation of encapsulated stem cells [186]. Furthermore, such modifications could be readily modified to produce gradients of mechanical properties suitable for different layers of alveolar bone [187]. Additionally, to make bioprinted constructs clinically relevant for implantation strategies, incorporation of thermoplastic polymers such as poly-lactic co glycolic acid (PLGA) [188], polycaprolactone (PCL) or polyethelyne glycol (PEG), acting as support structures with tunable mechanical properties for the hydrogel, is necessary to ensure their long-term preservation, especially in load bearing areas of the alveolar bone. Moreover, to enhance the ability for hard tissue regeneration in larger defects, angiogenesis is a critical aspect which could be achieved through incorporation of sacrificial layers with materials such as Pluronic F-127 [39], or with other strategies mentioned in the review such as the incorporation of endothelial cells or hypoxia conditioning.

While the application of bioprinting has presented a clear path towards the engineering of complex structures in the dentoalveolar region, the research still has to take advantage of more advanced bioprinting techniques which increase the resolution of the printing and enable bioprinting of fine structures such as capillary networks, nerves, or tissue-specific compositions such as dentin tubules. Coaxial bioprinting is one of these approaches, featuring multimaterial bioprinting and enabling bioprinting of complex structures such as hollow tubes with variable inner and outer diameters [189]. Bioprinting using support baths, or freeform bioprinting, is another approach that enables fabrication of constructs without the need for support layers, leading to the possibility of bioprinting irregular geometries observed in the region [190]. Moreover, emerging techniques such as volumetric bioprinting significantly increase both the speed and the precision of the bioprinting of complex tissues [191].

## 5. Conclusions

Regenerative medicine has opened up new horizons for the field of dentoalveolar treatments when traditional treatments are faced with shortcomings. Yet, the clinical translation of tissue regeneration strategies is hitherto still limited. Some of the hurdles include the failure of recapitulating complex tissue architectures as well as the variability in the process. These limitations often lead to a biological outcome that is not clinically relevant. However, with bioprinting, significant hope has been generated that some of these challenges could be overcome. Still, one of the main challenges in the field of bioprinting dentoalveolar tissues is making an appropriate choice of materials that support the encapsulation of cells and should be compatible with the bioprinting process. With the current research on bioprinting dentoalveolar tissue being in its early stages, there is still a wide range of possibilities for further research. To provide a solid basis for future studies, this review summarized the results obtained in this area thus far, and provided some future perspectives on how the field could move forward to improve dentoalveolar tissue regeneration using bioprinting strategies.

## Figures and Tables

**Table 1:** Overview of the research on bioprinting of dental pulp, periodontal ligament and dentoalveolar bone published in English up to November 2021.

Tissue	Bioprinting Strategy	Material Used	Nozzle Size	Study Type	Ref.
alveolar bone	Stereolitography	gelatin methacrylate	-	*In vitro*	Amler et al. 2021 [32]
alveolar bone	Stereolitography	gelatin methacrylate + methacrylated hyaluronic acid	-	*In vitro*	Amler et al. 2021 [33]
alveolar bone	Extrusion	β-TCP + nanofibrillated cellulose/alginate	Coaxial: ≈406–535 µm (22–18 G) ≈406–885 µm (22–16 G) ≈406–1295 µm (22–14 G)	*In vitro*	Walladbegi et al. 2020 [34]
alveolar bone	Inkjet	ECM-based hydrogel + self-assembling FEFEFKFK octapeptide + amorphous magnesium phosphates	-	*In vitro + In vivo*	Dubey et al. 2020 [35]
alveolar bone	Extrusion	gelMA + kappa-carrageenan + nanosilicates	400 µm (≈22 G)	*In vitro*	Chimene et al. 2020 [36]
alveolar bone	Inkjet	gelatin methacrylate + poly (ethylene glycol)	150 µm (≈30 G)	*In vitro + In vivo*	Ma et al. 2017 [37]
alveolar bone	Extrusion	methacrylated hyaluronic acid + gelatin methacrylate	400 µm (≈22 G)	*In vitro*	Kuss et al. 2017 [38]
alveolar bone	Extrusion	gelatin + hyaluronic acid + fibrinogen + glycerol	300 µm (≈23 G)	*In vivo*	Kang et al. 2016 [39]
alveolar bone	Extrusion	collagen type I + agarose	600 µm (≈20 G)	*In vitro*	Campos et al. 2016 [40]
bone/alveolar bone	Extrusion	sodium alginate + gelatin + nano-hydroxyapatite	400 µm (≈22 G)	*In vitro*	Tian et al. 2020 [41]
periodontal ligament	In-house—single-cell printing	-	≈240 µm (26 G)	*In vitro*	Tomokiyo et al. 2021 [42]
periodontal ligament	Extrusion	collagen + FGF-2	400 µm (≈22 G)	*In vitro + In vivo*	Lee et al. 2021 [43]
periodontal ligament	Extrusion	gelatin methacrylate	≈220 µm (25 or 27 G)	*In vitro*	Raveendran et al. 2019 [44]
periodontal ligament	Inkjet	gelatin methacrylate + poly (ethylene glycol) dimethacrylate	150 µm (≈30 G)	*In vitro*	Ma et al. 2015 [45]
dentin pulp	Extrusion	Calcium silicate reinforced gelatin methacrylate	30 G (≈150 µm)	*In vitro*	Lin et al. 2021 [46]
dentin pulp	Extrusion	Fibrinogen—gelatin—demineralized dentin matrix particles	300 µm (≈23 G)	*In vitro*	Han et al. 2021 [47]
dentin pulp	Extrusion	fibrinogen + gelatin + hyaluronic acid + glycerol	300 µm (≈23 G)	*In vitro*	Han et al. 2019 [48]
dentin pulp	Extrusion	alginate + dentin matrix	≈450 µm (22 G)	*In vitro*	Athirasala et al. 2018 [49]
dental pulp	Extrusion	BMP-mimetic peptide modified GelMA + gelatin + hyaluronic acid + glycerol	330 µm (≈23 G)	*In vitro*	Park et al. 2020 [50]
dental pulp	Inkjet	agarose + collagen type I + fibrinogen	300 µm (≈23 G)	*In vitro*	Campos et al. 2019 [51]
dental pulp/cornea/articular cartilage	Inkjet	agarose + collagen type I	300 µm (≈23 G)	*In vitro*	Betsch et al. 2018 [52]

**Table 2:** An overview of some candidate materials with potential to be used in bioprinting for pulp-dentin, along with cell types and biological cues useful for the purpose. (IJ: inkjet, EX: extrusion. L: laser based).

Ref.	Material	Targeted Tissue	Cell Types Used	Bioprinted? (Tech)	Max Cell Viability (%)	Study Duration (Days)	*In Vivo*?	Suggestive Tissue	Suggestive Cell Types	Suggestive Biological Cues
[119]	alginate + matrigel + bioactive glass microparticles	pulp-dentin	dental pulp stem cells	No	80	21	✕	pulp-dentin	dental pulp stem cells (DPSCs) [131,132]/stem cells from apical papilla (SCAPs) [133]/human umbilical vein endothelial cells (HUVECs) [134]/odontoblast-like cells/stem cells from human exfoliated deciduous teeth	vascular endothelial growth factor (VEGF)/nerve growth factor (NGF)/bone morphogenetic protein 7 (BMP-7)/platelet-derived growth factor (PDGF)
[118]	RGD modified alginate	pulp-dentin	human umbilical vascular endothelial cells + human dental pulp stem cells	No	N/A	14	✕			
[114]	fibrin + polyethylene glycol	pulp-dentin	dental pulp stem cells	No	85	7	✕			
[107]	hyaluronic acid + cellulose nanocrystals + platelet lysate	pulp-dentin	dental pulp stem cell	No	N/A	14	✕			
[135]	gelatin norbornene + thyiolated gelatin	vascularized cardiac tissue	human umbilical vein endothelial cells + iPSC-derived cardiomyocytes	L	94	7	✕			
[136]	gelMA + gelatin + glycerol + hyaluronic acid	small blood vessels	human umbilical vein endothelial cells + smooth muscle cells	Ex	89.8	7	✕			
[137]	alginate + type I collagen	microvasculature	human umbilical vein endothelial cells	IJ	N/A	3	✓			
[138]	RGD modified elastin-like protein hydrogel	neural tissue model	neural progenitor cells + human induced pluripotent stem cells + human umbilical vein endothelial cells + human premalignant breast epithelial cells	IJ	88.3	14	✕			
[139]	alginate in nanoclay support bath	complex vascular structures	NIH/3T3 fibroblasts	Ex	94.3	7	✕			
[140]	alginate + gelatin + carbon nanotubes	vessel constructs	fibroblasts	Ex	86.6	7	✕			
[141]	alginate-methylcellulose	bioinks for gene delivery	bone marrow-derived mesenchymal stem cells	Ex	N/A	N/A	✓			
[142]	collagen type I	capillary network	stem cells from the apical papilla	L	N/A	N/A	✕			

**Table 3:** An overview of some candidate materials with potential to be used in bioprinting for periodontal ligament and alveolar bone, along with cell types and biological cues useful for the purpose. (IJ: inkjet, EX: extrusion. L: laser based).

Ref.	Material	Targeted Tissue	Cell Types Used	Bioprinted? (Tech)	Max Cell Viability (%)	Study Duration (Days)	*In Vivo*?	Suggestive Tissue	Suggestive Cell Types	Suggestive Biological Cues
[143]	alginate + sodium periodate	periodontal ligament	periodontal ligament stem cells + gingival mesenchymal stem cells	No	95	28	✓	periodontal ligament	periodontal ligament stem cells (PDLSCs) [144]/gingival mesenchymal stem cells [145]	connective tissuee growth factor (CTGF) + transforming growth factor-β3 (TGF-β3) [146]/transforming growth factor B1 (TGFB1)
[147]	gelatin + fibrinogen + hyaluronic acid + glycerol + PCL support	anisotropic cartilage	bone marrow stromal cell	Ex	75	21	✓			
[146]	pluronic + alginate	liver model	hepG2/C3A cell line	Ex	N/A	7	✕			
[148]	decellularized tendon extracellular matrix	tendon tissue	NIH 3T3 cells	Ex	≈ 85	3	✕			
[149]	methacryloyl-polyethylenglycol dimethacrylate	muscle and tendon tissues	primary human skeletal-muscle-derived cells + Primary rat tail tenocytes	IJ	95	<1	✕			
[150]	hyaluronic acid + gelatin + fibrinogen + polyurethane support	muscle tendon unit	C2C12 cell line + NIH/3T3 cell line	Ex	80	7	✕			
[151]	gelMA, collagen methacrylate, fibronectin, laminin	cardiac muscle	human-induced pluripotent stem cells	Ex	N/A	13	✕			
[152]	collagen + fibrinogen + alginate	multilayered vascular tissue constructs	human umbilical vein endothelial cells	Ex	N/A	5	✕			
[153]	chitosan + chitosan-hyaluronic acid	bone tissue	MC3T3-E1 pre-osteoblast cell line	Ex	95	9	✕	Alveolar bone	dental pulp stem cells (DPSCs) [154]/human umbilical vein endothelial cells (HUVECs) [155]/bone marrow mesenchymal stem cells (BMSCs) [156]/osteoblast cell precursor MC3T3-E1 [157]	periostin + TGF-β [158] transforming growth factor–β3 (TGFβ3), bone + morphogenetic protein 4 (BMP4) [147]/basic fibroblast growth factor (bFGF)/vascular endothelial growth factor (VEGF)
[106]	hyaluronic acid + polycaprolactone	alveolar bone	osteoblasts	No	75	7	✓			
[159]	gelatin + hyaluronic acid + fibrinogen + glycerol + hydroxyapatite + aprotinin	prevascularized bone tissue	human adipose-derived mesenchymal stem cells + human umbilical vein endothelial cells	IJ	90	<1	✓			
[160]	alginate + gelatin + glycerol	bone tissue	human mesenchymal stem cells	Ex	≈85	7	✕			
[161]	Gelatin—ureido-pyrimidinone—tyramine	complex structures	human bone marrow mesenchymal stem cell + endothelial cells +	Ex	90	1	✕			
[56]	oligo(poly[ethylene glycol] fumarate) + gelatin	bone and nerve	MC3T3-E1 pre-osteoblast cells	Ex	N/A	7	✕			
[162]	blood plasma + alginate + methylcellulose + calcium phosphate cement support	bone tissue	mesenchymal stem cells	Ex	75	<1	✕			
[163]	collagen + β-Tricalcium phosphate	bone tissue	MC3T3-E1 pre-osteoblast cells + human adipose stem cells	Ex	92	<1	✕			
[164]	poly(ethylene glycol) dimethacrylate + acrylated GRGDS and MMP-sensitive peptides	bone and cartilage	human mesenchymal stem cells	IJ	≈88	1	✕			
[165]	alginate + methylcellulose + laponite	bone tissue	immortalised human mesenchymal stem cells	Ex	75	21	✕			
[166]	carboxymethyl chitosan + amorphous calcium phosphate	bone tissue	mesenchymal stem cell	Ex	N/A	15	✓			

## Data Availability

Data sharing is not applicable to this article.
